# Emerging Technologies in Endocrine Drug Delivery: Innovations for Improved Patient Care

**DOI:** 10.7759/cureus.62324

**Published:** 2024-06-13

**Authors:** Mahvish Renzu, Carly Hubers, Kendall Conway, Viktoriya Gibatova, Vidhi Mehta, Wael Taha

**Affiliations:** 1 Internal Medicine, Trinity Health Oakland/Wayne State University, Pontiac, USA; 2 Internal Medicine, School of Medicine, Wayne State University, Detroit, USA; 3 Internal Medicine, Ross University School of Medicine, Miramar, USA; 4 Endocrinology, Trinity Health, Mercy, USA; 5 Endocrinology, Detroit Medical Center/Wayne State University, Detroit, USA

**Keywords:** endocrine drug delivery, inhaled insulin, artificial pancreas, emerging technologies, endocrine treatments

## Abstract

Recent advancements in drug delivery systems for endocrine disorders have significantly improved patient outcomes by addressing the limitations of traditional methods such as oral tablets and injections. These innovations include non-invasive alternatives like inhaled insulin, which provides rapid absorption and better patient compliance, and robotic pills that deliver drugs directly to specific gastrointestinal sites, enhancing absorption and reducing side effects.

Wearable artificial pancreas systems have revolutionized diabetes management by integrating continuous glucose monitoring with insulin pumps to automate blood glucose control. These systems demonstrate superior glycemic control and reduce hypoglycemic events. Additionally, smart insulin pens enhance diabetes care through dose tracking and real-time data sharing, improving accuracy and adherence.

Microneedle patches offer a minimally invasive method for transdermal drug delivery, effectively administering hormones and therapeutic peptides without the pain and inconvenience of injections. These patches dissolve after use, eliminating biohazardous waste. Implantable devices provide long-term, controlled release of medications, significantly improving adherence and glycemic control of patients with diabetes. Hydrogels also offer new drug delivery options.

This review examines these technologies' clinical efficacy, safety, advantages, and limitations, highlighting their potential to transform endocrine disorder management. Integrating advanced delivery systems marks a significant step towards personalized medicine, tailoring treatments to individual patient needs for better health outcomes.

## Introduction and background

Challenges in traditional drug delivery

The delivery of therapeutic agents in the management of endocrine disorders, which include conditions like diabetes and thyroid diseases, has seen significant advancements over the past decade. Traditional methods of drug administration, such as oral tablets and injections, often pose challenges in terms of patient adherence, drug stability, and targeted delivery. Oral tablets require patients to remember to take their medication regularly, which can be inconvenient and easy to forget, while injections can be painful and intimidating for many individuals. These challenges highlight the need for more effective and user-friendly delivery methods. As a result, the development of innovative drug delivery systems has been spurred, aimed at enhancing the efficacy, safety, and convenience of endocrine therapies. These new technologies not only improve how drugs are delivered and absorbed in the body but also aim to reduce the burden on patients. They have the potential to improve patient's adherence to treatment and overall quality of life.

Emerging solutions

Notable advancements include the development of inhaled insulin, which offers a non-invasive alternative to subcutaneous injections for diabetes management. Inhaled insulin has been shown to provide rapid absorption. Similarly, robotic pills (RPs) represent a groundbreaking innovation in oral drug delivery. These ingestible devices can deliver drugs directly to specific sites in the gastrointestinal (GI) tract, enhancing absorption and reducing systemic side effects. Wearable artificial pancreas systems represent another significant leap forward in diabetes management. Another alternative is the advent of smart insulin pens, which track insulin doses and provide real-time data. This is revolutionizing diabetes care by improving accuracy and patient engagement. More promising technology includes the use of microneedle patches, which enable painless and efficient transdermal delivery of hormones. These patches are designed to enhance patient comfort and compliance, offering a viable alternative to traditional injections. Furthermore, implantable drug delivery devices provide long-term, continuous release of medications, thereby improving adherence and therapeutic outcomes. Hydrogels, with their highly tunable physical properties and ability to provide controlled drug release, have become a promising technology as well for enhancing the delivery and efficacy of therapeutic agents in endocrine disorder management. These technological innovations not only improve the pharmacokinetics and pharmacodynamics of endocrine therapies but also enhance patient adherence and quality of life.

Scope of review

This review aims to provide a comprehensive overview of the recent advancements in endocrine drug delivery technologies, including inhaled insulin, peptide-based drugs, RPs, implantable devices, wearable artificial pancreas systems, smart insulin pens, microneedle patches, and hydrogels. By examining how effective and safe these new methods are, along with their benefits and limitations, we hope to show how they can transform the treatment of endocrine disorders and improve patient outcomes.

To ensure replicability and reproducibility in alignment with systematic review standards, we have explained our search approach. We conducted a comprehensive search across multiple databases, including PubMed, Google Scholar, and Scopus. Some specific search terms and Boolean operators used in our search included “endocrine drug delivery,” “inhaled insulin,” “artificial pancreas,” “emerging technologies,” “microneedle patches,” “implantable devices,” “hydrogels,” and “smart insulin pens.” The search was focused on literature published within the last 10 years to capture the most recent advancements, except for a few older sources for historical significance. We did not impose any language restrictions, ensuring a broad inclusion of relevant studies. Additionally, we reviewed other sources such as conference proceedings and unpublished works to ensure a comprehensive overview of the current state of research in this field. This detailed search strategy enhances the transparency and rigor of our review.

## Review

Inhaled insulin

In 1921, insulin was introduced by Banting and Best from the University of Toronto as an injectable agent. Before the advent of recombinant DNA technology allowed for the synthesis of human insulin (humulin), it was derived from animal sources such as beef and porcine insulin [1]. Today, several innovative technologies are emerging in the field of insulin delivery aimed at making treatment more convenient and effective, such as inhaled insulin. The idea of inhalable insulin was first proposed by German researchers in 1924, but it took many years and technological advancements to make it a reality. In the 1980s, Nektar Therapeutics developed a method to convert insulin into small particles suitable for inhalation, eventually licensing the technology to Pfizer. Human trials for inhalable insulin began in the late 1990s. Then, in 2006, the FDA approved Exubera (Nektar Therapeutics, San Francisco, CA), the first inhalable insulin, though it failed to gain widespread acceptance among patients and physicians. However, in June 2014, the FDA approved Afrezza (MannKind Corporation, Danbury, CT) for use in both Type I and Type II diabetics, marking a significant milestone in the development of non-invasive insulin delivery systems [2]. Inhaled insulin systems such as those developed by Nektar Therapeutics and Pfizer have been shown to reach the bloodstream faster than subcutaneous insulin, making them an ideal solution for postprandial glycemic control​. Afrezza offers a rapid-acting, convenient alternative to injections, though its use is restricted in patients with asthma, active lung cancer, or chronic obstructive pulmonary disease. 

The uses of Afrezza are still being explored in various clinical settings to fully understand its potential benefits and applications. The INHALE-3 study, produced by MannKind Corporation, focuses on the impact of Afrezza Inhalation Powder on mealtime glucose control. Afrezza is classified under the anatomical therapeutic chemical (ATC) classification system as A10AB06, indicating it is an insulin and analog for injection, fast-acting. The INHALE-3 study is a Phase 4 randomized controlled trial evaluating the efficacy and safety of inhaled insulin Afrezza combined with insulin Degludec (Novo Nordisk, Bagsværd, Denmark) versus usual care in adults with Type I diabetes. This trial involves 120 participants who were randomized to either the Afrezza-Degludec regimen with continuous glucose monitoring (CGM) or their usual insulin regimen with CGM. The primary outcome is assessed at 17 weeks, followed by a 13-week extension phase where both groups use the Degludec-Afrezza regimen [3]. Early results indicated that the combination of Degludec and Afrezza may improve postprandial glucose control and provide benefits in secondary glucometric endpoints, such as time in range and time below range. Participants in the control group were given the option to switch to the Afrezza regimen during the extension phase, offering additional insights into patient preferences. Subgroup analyses are exploring the effects of the therapy in different patient populations, including those using multiple daily injections and automated insulin delivery systems [3]. While full results are awaited, this combination therapy could offer a viable alternative to traditional insulin regimens, potentially improving glycemic control and quality of life.

Peptide-based drugs

**Figure 1:**
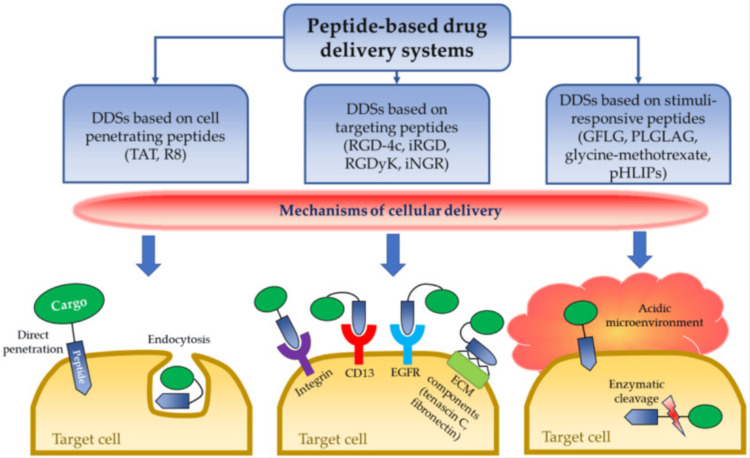
Peptide-based drug delivery systems Source: Ref. [4] (CC BY 4.0).

Peptide-based drugs are becoming increasingly significant in endocrinology due to their ability to mimic natural hormones and offer targeted therapeutic effects (Figure 1). One notable example is glucagon-like peptide-1 (GLP-1) receptor agonists, such as exenatide. These drugs enhance insulin secretion, suppress glucagon release, slow gastric emptying, and promote satiety, making them effective for managing type 2 diabetes and obesity. Exenatide (Amylin Pharmaceuticals, Inc., San Diego, CA), derived from exendin-4, has demonstrated significant benefits in lowering blood glucose levels and promoting weight loss in both animal models and human studies​ [4]. 

Another innovative development in peptide-based drug delivery is the NIPEP-TPP technology, which enhances the oral and targeted tissue delivery of therapeutic peptides. This technology involves cell-penetrating peptides that can transport therapeutic agents across cellular barriers, including the blood-brain barrier, making it promising for treating central nervous system disorders. Additionally, this method can protect peptides from enzymatic degradation in the GI tract, improving the efficacy of oral peptide drugs like GLP-1 receptor agonists​ [5]. These advancements in peptide-based therapies and delivery methods are paving the way for more effective treatments in endocrinology.

Robotic pill

**Figure 2:**
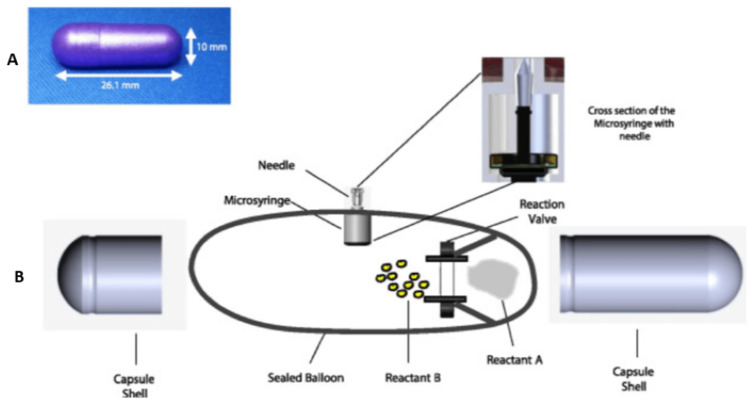
Robotic pill (RP) design (A) Fully assembled enteric-coated RP. (B) Schematic drawing showing various parts and components of the RP. Inset shows the microsyringe containing the needle with the drug microtablet which gets injected into the jejunal wall. The microtablet and needle are aseptically manufactured in an isolator and hermetically sealed inside a drug chamber which is then inserted in the microsyringe. Source: Ref. [6] (CC BY 4.0).

Biotherapeutics have proven to be highly efficacious. Today, over 350 biologics are on the market for treating various diseases [6]. However, biotherapeutics must be injected parenterally due to their susceptibility to digestion, which burdens patients and can lead to adverse events from injections. Consequently, achieving the convenience of oral delivery of biotherapeutics is highly desirable to enhance patient compliance and therapeutic outcomes. To address this challenge, an orally ingestible RP has been developed, which protects the biotherapeutic drug payload from digestion in the GI tract and auto-injects it into the wall of the small intestine. The novel RP for drug delivery has been designed to deliver various biotherapeutics for multiple indications. This method provides a safe, pain-free injection since the intestines are insensate to sharp stimuli. Enclosed in a standard pharmaceutical capsule shell, the RP is enteric coated to prevent its dissolution in the acidic stomach environment (Figures 2A, 2B). Upon reaching the small intestine, the pH change dissolves the enteric coating, triggering a chemical reaction that rapidly inflates a balloon within the RP. This aligns a microsyringe to inject the drug payload into the intestinal wall painlessly. Preclinical studies have shown that the RP can deliver biotherapeutics with high bioavailability in porcine and canine models [6]. The RP, essentially a swallowable auto-injector, has successfully demonstrated its ability to deliver drugs safely and efficiently, providing high bioavailability in a simple oral pill form. Specifically, the RP has shown its favorable application in the administration of synthetic hormones, such as teriparatide. Sold under the brand name Forteo (Eli Lilly and Company, Indianapolis, IN) teriparatide is a synthetic analog of human parathyroid hormone (PTH) intended for the treatment of osteoporosis in individuals at high risk of fractures. It is currently available exclusively as an injectable formulation, which is not ideal. 

A Phase I study conducted by Myers et al., involving 29 healthy female volunteers, assessed the viability of teriparatide delivery through RP as a potential alternative. Pharmacokinetics of teriparatide administration were compared using the RP versus standard subcutaneous delivery. The RP demonstrated encouraging results achieving bioavailability that was three to fourfold higher compared to standard methods. Additionally, there were no serious adverse events identified with the administration and subsequent excretion of RP device remnants, allowing RP to safely deliver teriparatide with high reliability [7]. In another Phase I trial involving 39 fit women, the RP successfully administered varying doses of teriparatide, once again demonstrating its effectiveness compared to conventional injections. Researchers used fluoroscopic imaging to track the pill's movement within the body and collected blood samples to measure drug concentration, ensuring precise insights into its performance [8]. Other clinical studies in healthy human subjects have further demonstrated the RP's safety, tolerability, and performance. One such study tracked the bioavailability of octreotide, an injectable therapeutic peptide, while another assessed the RP's deployment and the effect of food [6]. These advancements in RP technology represent a significant step towards the successful oral delivery of biotherapeutics, potentially transforming patient care. 

Implantable drug delivery devices

**Figure 3:**
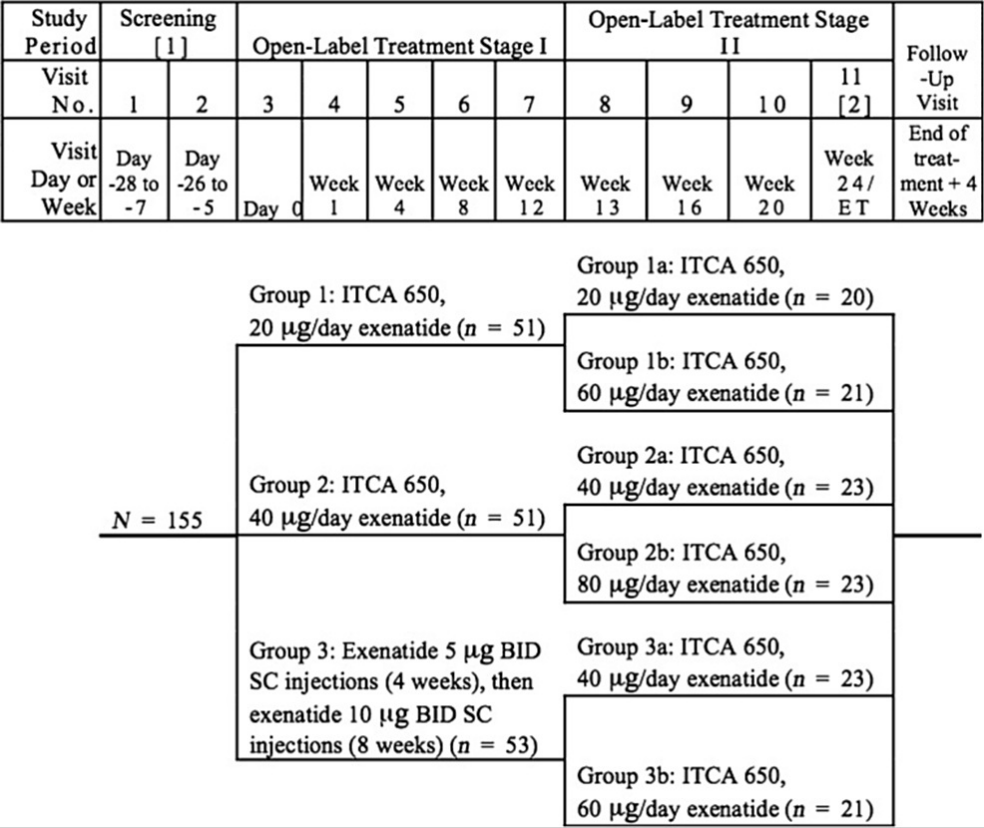
Study flow chart Source: Ref. [9]. Available under the Creative Commons Attribution-NonCommercial-NoDerivatives 3.0 Unported License

Implantable drug delivery devices represent another significant advancement in the management of chronic conditions, particularly for patients with Type II diabetes. In November 2016, Intarcia Therapeutics, Inc. submitted a new drug application for ITCA 650, the first implanted, injection-free GLP-1 receptor agonist delivery device. This innovative device dispenses exenatide through an osmotic mini pump for up to one year, providing a consistent and controlled release of medication [9]. ITCA 650 (Intarcia Therapeutics, Inc., Boston, MA) is designed for patients who have inadequate glycemic control from diet, exercise, and other antidiabetic medications. In clinical trials (Figure 3), ITCA 650 has demonstrated significant improvements in glycemic control and weight reduction, offering a dual benefit for patients with Type II diabetes [9].

The extended duration of ITCA 650's delivery system addresses a critical challenge in diabetes management: medication adherence. Many patients struggle with the daily or weekly injections required by current GLP-1 receptor agonists, whereas ITCA 650 offers a more convenient alternative. In a 24-week trial, 85% of eligible participants opted to continue using ITCA 650 in an additional 24-week extension phase, indicating high tolerability and patient preference for this delivery method [10]. Furthermore, comprehensive insurance coverage for the placement, maintenance, and removal of nonbiodegradable drug delivery systems like ITCA 650 can significantly reduce direct patient costs. It also aids in the proper pharmacoeconomic evaluation of diabetes management. This support makes ITCA 650 a more cost-effective option for long-term diabetes management, enhancing accessibility and affordability for patients.

Wearable artificial pancreas systems

**Figure 4:**
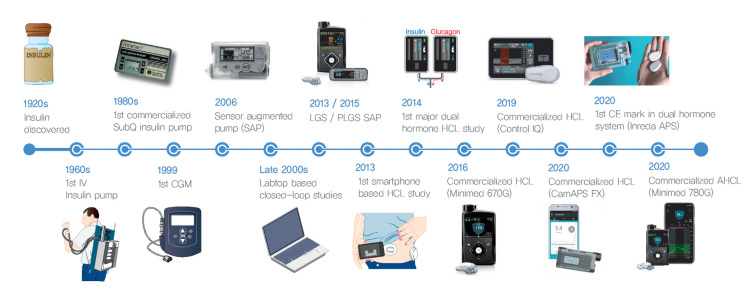
Timeline of development of the artificial pancreas system IV, intravenous; SubQ, subcutaneous; CGM, continuous glucose monitoring system; SAP, sensor augmented pump; LGS, low glucose suspension; PLGS, predictive low glucose suspension; HCL, hybrid closed-loop; CE, Conformité Européenne; APS, artificial pancreas system; AHCL, advanced hybrid closed-loop. Source: Ref. [11] (CC BY-NC 4.0).

Wearable artificial pancreas systems represent a significant advancement in the management of diabetes, especially for patients with Type I diabetes (Figure 4).

**Figure 5:**
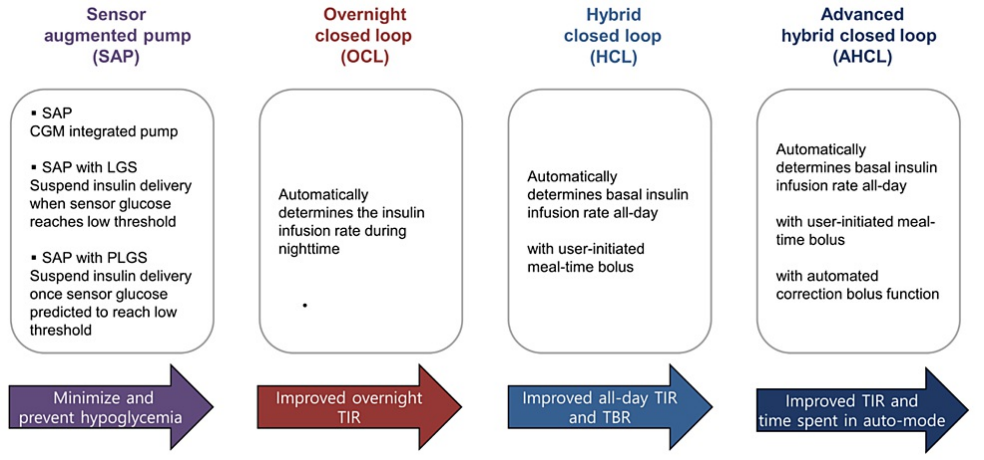
Key features of sensor augmented pump and artificial pancreas systems CGM, continuous glucose monitoring system; LGS, low glucose suspension; PLGS, predictive low glucose suspension; TIR, time in range; TBR, time below range. Source: Ref. [11] (CC BY-NC 4.0).

These systems integrate CGM with insulin pumps to create a closed-loop system that automates insulin delivery (Figure 5).

The primary goal of these systems is to maintain blood glucose levels within a target range, reducing the burden on patients to manage their condition manually. Various brands and types of wearable artificial pancreas systems are available commercially, each offering unique features to help manage diabetes more effectively. Some of the leading systems include the Medtronic MiniMed 780G (Medtronic, Dublin, Ireland), Tandem Diabetes Care's t:slim X2 with Control-IQ (Tandem Diabetes Care, Inc., San Diego, CA), and the Omnipod 5 Automated Insulin Delivery System (Insulet Corporation, Acton, MA). These devices integrate CGM with advanced insulin pumps to automate blood glucose control, providing patients with a more convenient and efficient way to manage their condition. Each system employs sophisticated algorithms and biometric data to tailor insulin delivery to the individual's needs, aiming to maintain optimal glucose levels.

The clinical effectiveness of these devices has been validated through numerous studies showing their superiority over traditional methods. For instance, research has indicated that the use of an automated pancreas system can improve overall glucose control with fewer hypoglycemic events, enhancing the quality of life for individuals with Type I diabetes [11]. The use of biometric variables, such as heart rate and skin temperature, have been shown to enhance the performance of these systems by providing additional data that can predict glucose fluctuations due to factors like exercise and stress. Research has indicated that using heart rate information alongside CGM data can improve the system's ability to manage glucose levels during physical activity. This enhancement is crucial as it allows patients to engage in physical activities with a reduced risk of dangerous drops in blood sugar levels. Furthermore, advancements in sensor technology and machine learning algorithms continue to refine these systems, making them more reliable and user-friendly, ultimately improving the quality of life for diabetes patients [12].

Smart insulin pens

Smart insulin pens, such as the InPen (Medtronic, Dublin, Ireland) and NovoPen 6 (Novo Nordisk, Bagsværd, Denmark), represent a big advancement in diabetes management by integrating technology to improve the accuracy and convenience of insulin administration. These pens offer features like dose tracking, reminders, and connectivity to mobile apps, which help users manage their insulin therapy more effectively. By recording each dose and providing real-time data, smart insulin pens address common issues related to missed doses and dosing errors, thereby enhancing patient adherence and glycemic control. Studies have shown that these devices not only improve patient confidence in managing their condition, but also facilitate better communication with healthcare providers through data sharing. This can lead to more personalized and effective diabetes care [13]. 

Microneedle patches

**Figure 6:**
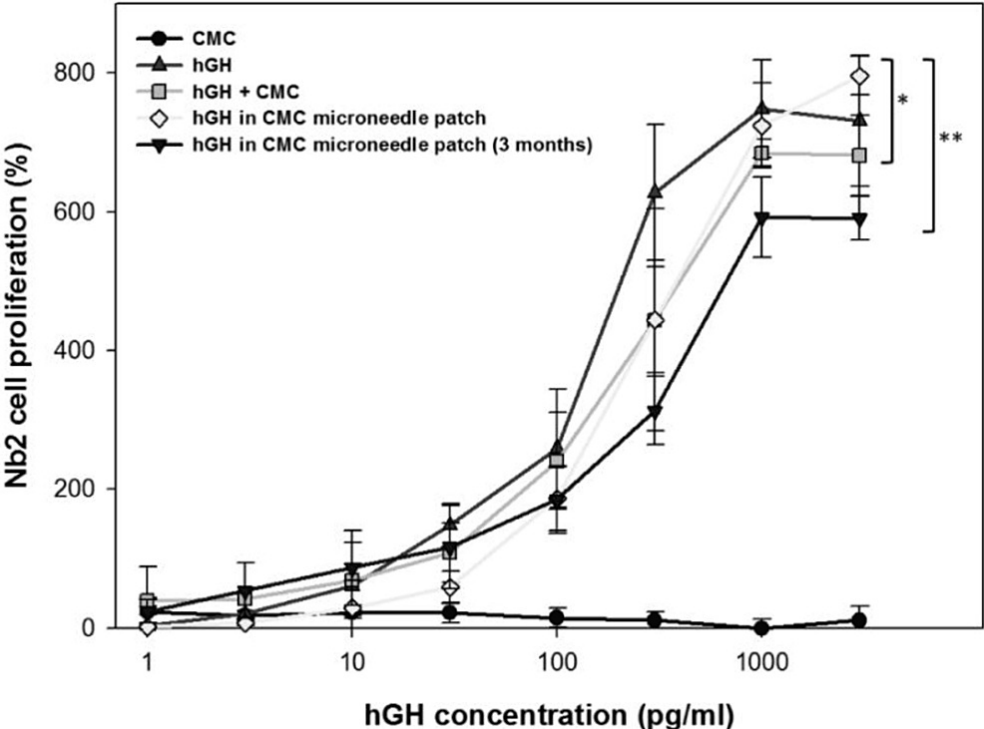
Human growth hormone (hGH) stability after encapsulation in a dissolving microneedle patch The addition of hGH to Nb2 cell culture in the stationary phase stimulated the proliferation of Nb2 cells, which was recorded at three days after hGH treatment and used as a measure of the functional activity of hGH after encapsulation in dissolving microneedles. Five experimental groups were studied: CMC solution by the reconstitution of a placebo CMC microneedle patch (CMC, negative control, ●), hGH solution (hGH, positive control, ▲), hGH solution mixed with CMC reconstituted from a placebo CMC microneedle patch (hGH + CMC, ▪), hGH and CMC solution reconstituted from a CMC microneedle patch encapsulating hGH (hGH in CMC microneedle patch, ◆), and hGH and CMC solution reconstituted from a CMC microneedle patch encapsulating hGH after storage for three months at ambient conditions (hGH in CMC microneedle patch (three months), ▾). All groups contain CMC and hGH at the same mass ratio (5 hGH: 95 CMC) at all hGH concentrations, except the positive control. Asterisk indicates comparison with the hGH positive control (two-way ANOVA, p > 0.05 for * and p < 0.05 for **). Data points represent the average ± standard deviation for n=4 replicates for all groups. Source: Ref. [14] (CC BY-NC 3.0).

Microneedle patches have emerged as a promising technology for the transdermal delivery of biopharmaceuticals, providing a minimally invasive alternative to traditional hypodermic injections. These patches consist of micron-scale needles that penetrate the outermost layer of the skin, allowing for the direct delivery of drugs into the bloodstream. This method is particularly advantageous for the administration of proteins and peptides, which are ineffective if delivered orally due to their degradation in the GI tract. One study demonstrated the use of dissolving microneedle patches for the transdermal delivery of human growth hormone (Figure 6). It showed that the pharmacokinetics were similar to conventional subcutaneous injections and that the microneedles dissolved completely after use, leaving no sharp biohazardous waste behind [14]. 

**Figure 7:**
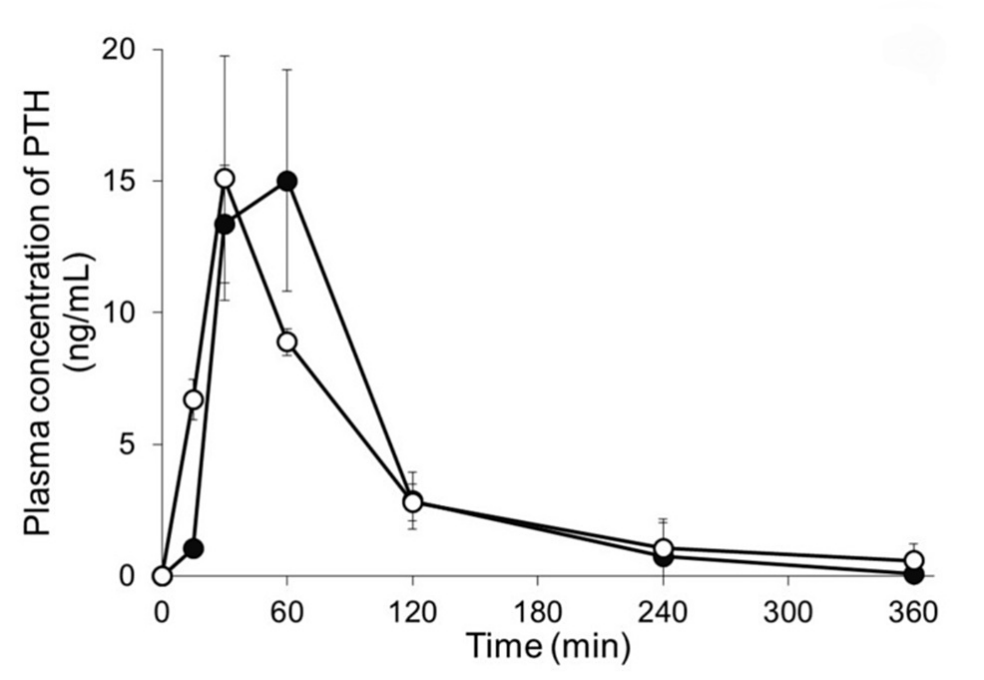
Plasma concentration-time profiles of human parathyroid hormone Plasma concentration-time profiles of human parathyroid hormone (1-34) (PTH) following either application of microneedle arrays (MNs) or subcutaneous injection. Keys: (●) PTH-loaded MNs and (〇) subcutaneous injection of PTH. Results are expressed as the means ± standard deviation (SD) of three rats. Source: Ref. [16] (CC BY 4.0).

Further advancements have been made in the design of microneedle patches for the delivery of other therapeutic peptides. For instance, a study on the development of a hyaluronic acid-based microneedle patch for the delivery of PRL-2903, an antagonist of somatostatin receptor type 2, showed promising results in preventing hypoglycemia in a Type I diabetes rat model. This patch effectively increased plasma glucagon levels and restored blood glucose regulation, highlighting its potential for managing hypoglycemia alongside insulin therapy [15]. Additionally, research on self-dissolving microneedle arrays for the delivery of PTH demonstrated high bioavailability and efficacy in treating osteoporosis in rat models (Figure 7), further establishing the versatility and clinical potential of microneedle patches for various medical applications [16].

Hydrogel drug delivery 

A recent breakthrough in diabetes management has been achieved by materials engineers who have developed an innovative hydrogel drug delivery system. This new method aims to significantly reduce the frequency of GLP-1 drug injections required for patients with Type II diabetes. Hydrogels offer great advantages in protecting labile drugs from degradation, thus enhancing their therapeutic efficacy. For instance, supramolecular hydrogels, which utilize non-covalent interactions for cross-linking, can simultaneously achieve drug loading and gelation in an aqueous environment without requiring covalent bonds. This approach allows for high drug loading capacity and stability, making hydrogels a promising vehicle for sustained and controlled drug delivery in various medical treatments​​ [17].

Moreover, hydrogels can be designed to respond to specific environmental stimuli, such as pH, temperature, and glucose concentration, enabling precise and targeted drug release. This feature is beneficial in treating conditions like diabetes, where local physiological changes can trigger the release of therapeutic agents, thereby optimizing treatment outcomes and minimizing side effects​​ [17]. The hydrogel technology allows for the administration of GLP-1 drugs just once every four months, in stark contrast to the current daily or weekly injections. This advancement not only promises to enhance patient compliance, but also aims to improve long-term health outcomes by reducing the burden of frequent injections. The hydrogel functions through a polymer-nanoparticle mesh that gradually dissolves, releasing the drug consistently over several months. This method has shown high success in laboratory rats and holds the potential for broader applications in various medical treatments, offering a promising future for more convenient and effective diabetes management [18].

## Conclusions

These aforementioned advancements are all part of a broader trend toward personalized medicine, aiming to tailor treatments to individual patient needs and improve overall health outcomes in the endocrine specialty. The continued evolution of drug delivery technologies promises to address many of the existing challenges in endocrine disorder management, paving the way for better care. Future research and development should focus on optimizing these systems for broader accessibility and affordability, ensuring that more patients can benefit from these advancements. As these innovative solutions become more integrated into clinical practice, they hold the potential to significantly improve patient adherence, treatment efficacy, and overall quality of life for individuals with endocrine disorders.
